# Structural studies of the mechanism for biosensing antibiotics in a fluorescein-labeled *β*-lactamase

**DOI:** 10.1186/1472-6807-11-15

**Published:** 2011-03-28

**Authors:** Wai-Ting Wong, Ho-Wah Au, Hong-Kin Yap, Yun-Chung Leung, Kwok-Yin Wong, Yanxiang Zhao

**Affiliations:** 1 Department of Applied Biology and Chemical Technology, Central Laboratory of the Institute of Molecular Technology for Drug Discovery and Synthesis, The Hong Kong Polytechnic University, Hung Hom, Hong Hong, China

## Abstract

**Background:**

*β*-lactamase conjugated with environment-sensitive fluorescein molecule to residue 166 on the Ω-loop near its catalytic site is a highly effective biosensor for *β*-lactam antibiotics. Yet the molecular mechanism of such fluorescence-based biosensing is not well understood.

**Results:**

Here we report the crystal structure of a Class A *β*-lactamase PenP from *Bacillus licheniformis *749/C with fluorescein conjugated at residue 166 after E166C mutation, both in *apo *form (PenP-E166Cf) and in covalent complex form with cefotaxime (PenP-E166Cf-cefotaxime), to illustrate its biosensing mechanism. In the *apo *structure the fluorescein molecule partially occupies the antibiotic binding site and is highly dynamic. In the PenP-E166Cf-cefatoxime complex structure the binding and subsequent acylation of cefotaxime to PenP displaces fluorescein from its original location to avoid steric clash. Such displacement causes the well-folded Ω-loop to become fully flexible and the conjugated fluorescein molecule to relocate to a more solvent exposed environment, hence enhancing its fluorescence emission. Furthermore, the fully flexible Ω-loop enables the narrow-spectrum PenP enzyme to bind cefotaxime in a mode that resembles the extended-spectrum *β*-lactamase.

**Conclusions:**

Our structural studies indicate the biosensing mechanism of a fluorescein-labelled *β*-lactamase. Such findings confirm our previous proposal based on molecular modelling and provide useful information for the rational design of *β*-lactamase-based biosensor to detect the wide spectrum of *β*-lactam antibiotics. The observation of increased Ω-loop flexibility upon conjugation of fluorophore may have the potential to serve as a screening tool for novel *β*-lactamase inhibitors that target the Ω-loop and not the active site.

## Background

*β*-Lactamase is one of the major mechanisms of antibiotic resistance in bacteria. Enzymes of this family deactivate *β*-lactam antibiotics by hydrolyzing the conserved *β*-lactam moiety in the antibiotics and rendering them ineffective to bind to their target proteins, the penicillin-binding proteins (PBPs), which are essential for bacterial cell wall synthesis and survival [1,2]. Detailed mechanistic studies of these enzymes over the past decades have revealed a conserved mechanism of *β*-lactam hydrolysis that consists of two steps, the acylation step in which the *β*-lactam ring is "opened" and acylated to the side chain hydroxyl group of Ser70 through nucleophilic attack to form the enzyme-substrate acyl adduct ES*; followed by the deacylation step in which the ES* intermediate is hydrolyzed and released as E + P facilitated by Glu166 (residue numbering according to the most conserved Class A *β*-lactamases) [3].

The substrate profile of a *β*-lactamase in hydrolyzing diverse *β*-lactam antibiotics is strongly influenced by a structural element termed Ω-loop, a short stretch of residues on the surface of the *β*-lactamase structure that forms part of the outer part of the antibiotic binding site [4-9]. For narrow-spectrum *β*-lactamases such as the PenP used in this study and the clinically significant TEM-1 or SHV-1 enzymes, Ω-loop is tightly packed onto the enzyme active site through hydrophobic and electrostatic interactions with residues lining the catalytic site, posing as steric hindrance for binding of second- or third-generation antibiotics with bulky side chains attached onto the *β*-lactam nucleus. Many mutant strains of TEM- and SHV-like *β*-lactamases overcome this inefficiency and broaden their hydrolytic profile by acquiring mutations in the Ω-loop region to render this region more flexible to accommodate large-sized antibiotics [4-10]. Many extended-spectrum *β*-lactamases have significantly extended Ω-loop, resulting in an enlarged active site that readily binds to and hydrolyzes almost all antibiotics [11-13].

Exploiting the proximity of Ω-loop to the antibiotic binding site and its structural flexibility, we have successfully converted a *β*-lactamase PenPC from *Bacillus cereus *569/H into a biosensor for *β*-lactam antibiotics by mutating the catalytically critical residue Glu166 on the Ω-loop to cysteine and conjugating an environment-sensitive fluorescein molecule to its reactive side chain thiol group to form PenPC-E166Cf as reported in previous studies [14-16]. Fluorescein is an environment-sensitive fluorophore with suppressed fluorescence in a hydrophobic environment but fluoresces strongly in a polar aqueous environment [17]. The mutation of Glu166 to cysteine severely reduces the efficiency of the deacylation step of *β*-lactamase catalysis, rendering the enzyme to stall at the acylation step and form a stable ES* acyl adduct that enhances the fluorescence emission of the conjugated fluorescein [16]. We have speculated that the fluorescein molecule is positioned near the catalytic site so that the binding and subsequent acylation of *β*-lactam antibiotics would displace it to a more polar environment, enhancing its fluorescence intensity [16].

Here we report structural studies of fluorescein-conjugated PenP *β*-lactamase from *Bacillus licheniformis *749/C to validate our proposed biosensing mechanism. The structural findings suggest an important role of Ω-loop in the biosensing process, which will help the rational design of improved biosensors for *β*-lactam detection as well as for novel antibiotics discovery.

## Results and Discussion

### The biosensing profile of PenP-E166Cf

The biosensing profile of fluorescein conjugated PenP (PenP-E166Cf) for detecting *β*-lactam antibiotics have never been reported before. In our previous study, a highly similar enzyme, PenPC from *Bacillus cereus *569/H with 58% amino acid sequence identity to PenP, was successfully engineered into a biosensor using the same design scheme (PenPC-E166Cf) [15,16]. We chose to work with PenP in this study for the advantage of its easy propensity for crystallization, which would enable structural studies to understand its biosensing mechanism at atomic resolution. PenPC, on the other hand, has poor thermal stability and is difficult to crystallize. Because of the high sequence similarity between these two proteins, as well as the general sequence conservation among all Class A *β*-lactamase enzymes we expect that PenP can serve as a good model system to understand the biosensing mechanism of fluorescein-based biosensing.

**Figure 1:**
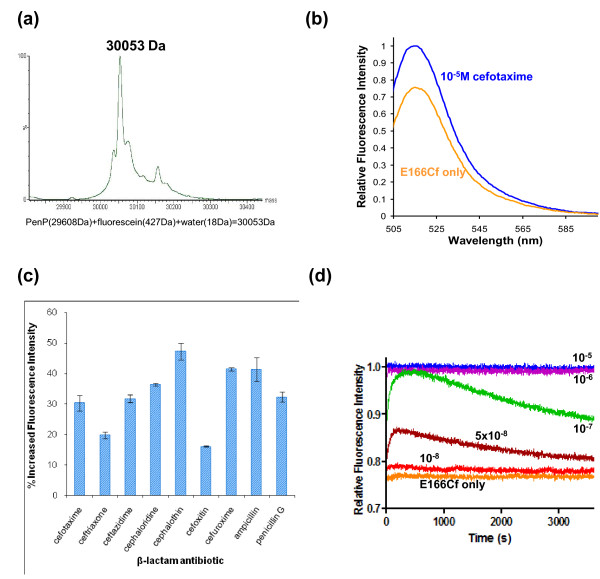
**Biosensing of β-lactam antibiotics by fluorescein-labelled PenP**. (a) De-convoluted ESI mass spectrum of PenP-E166Cf. The add-up at the bottom confirms the correct mass of the labelled protein. (b) Fluorescence scanning spectra of PenP-E166Cf in the presence of 10^-5^M cefotaxime in 50 mM phosphate buffer (pH 7.0). (c) Change in fluorescence emission of PenP-E166Cf after incubation with different antibiotics (cefotaxime, ceftriaxone, ceftazidime, cephaloridine, cephalothin, cefoxitin, cefuroxime, penicillin G and ampicillin) at 10^-6 ^M for 100 s. (d) Time-dependent fluorescence spectra in the presence of different concentrations (1 × 10^-8 ^M - 1 × 10^-5 ^M) of cefotaxime monitored at 515 nm.

Indeed the biosensing profile of PenP-E166Cf is highly similar to that of PenPC-E166Cf. The conjugation of fluorescein to the mutated Cys166 residue through thiol linkage is highly efficient for PenP. The ESI-MS profile confirmed that over 90% of PenP was labelled by the fluorophore and converted to PenP-E166Cf, with little unlabelled PenP remaining (Figure 1a). The fluorescence scanning spectrum of PenP-E166Cf shows an increase of ~25% in emitted intensity when the antibiotic cefotaxime is present at 10 μM concentration (Figure 1b). A variety of *β*-lactam antibiotics, including the first-, second- and third-generation compounds with diverse chemical structures in addition to the conserved *β*-lactam core, induce significant fluorescence enhancement in PenP-E166Cf at concentration as low as 1 μM (Figure 1c). Lastly the time-dependent spectra of PenP-E166Cf in the presence of cefotaxime at different concentrations ranging from 0.01 μM to 10 μM shows that PenP-E166Cf can detect cefotaxime at concentration as low as 0.01 μM and the fluorescence response is saturated at 1 μM (Figure 1d).

### The structure of PenP-E166Cf in *apo *form

PenP-E166Cf readily crystallized in the form of clustered needles. These crystals were tinted in bright yellow colour, indicating the presence of fluorescein (data not shown YZ). To confirm that fluorescein remaining conjugated to the protein in the crystal form we harvested and thoroughly washed these yellow-coloured crystals and analyzed the dissolved crystals on SDS-PAGE gel under both visible and UV light. A band corresponding to PenP (~30.5 kDa) is clearly visible under both conditions, confirming that the crystals are indeed of PenP-E166Cf (data not shown YZ).

**Figure 2:**
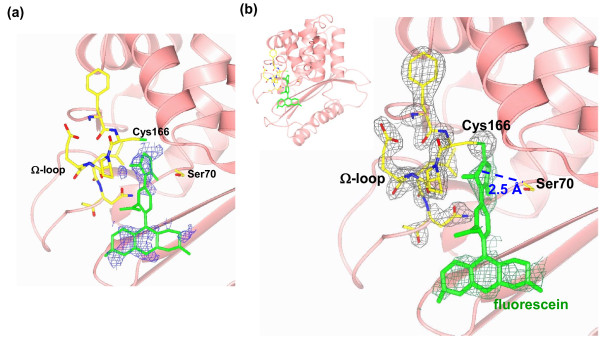
**Crystal structure of PenP-E166Cf**. (a) The *fo-fc *omit map of fluorescein-5-maleimide contoured at 2.0 σ. (b) The *2fo-fc *map of Phe165 to Asn170 and fluorescein-5-maleimide contoured at 1.0 σ. Side chains of Phe165 to Asn170 and Ser70 are shown in cpk cylinder model. Fluorescein is shown in green cylinder model.

The structure of PenP-E166Cf was solved by molecular replacement using the known structure of PenP (PDB ID 4BLM) as search model. Two molecules of PenP-E166Cf are found in each asymmetric unit. Structure rebuilding and refinement were done in CCP4 program [18]. The overall structure of PenP-E166Cf is largely identical to that of the wild-type unlabeled PenP. The RMSD of all 4011 protein atoms between the labeled and wild-type structures is just ~1.5 Å. For main chain atoms, the RMSD is only 0.8 Å. Key residues lining the catalytic site, including Ser70 and mutated Cys166 are virtually identical between the labeled and wild-type structures (Figure 2a). In summary the conjugation of fluorescein to PenP does not alter its overall structural folding.

The fluorescein molecule was modeled onto the PenP structure after careful inspection of the *fo-fc *and 2*fo-fc *electron density map. These maps are not of high quality at regions around the fluorescein conjugation site, with only pieces of discontinuous density visible at 2.0 σ contour level in the *fo-fc *map (Figure 2a). We tried our best to fit fluorescein into these pieces of electron density, particularly matching the melaimide group to a piece of electron density near the thiol side chain of Cys166, as well as matching the xanthene group at the end of the fluorescein molecule to a large piece of electron density near the catalytic site (Figure 2a). This modeled structure is stable after rounds of structural refinement, showing good electron density for the Ω-loop residues and the fluorescein molecule at 1.0 σ contour level in the *2fo-fc *map, suggesting that our fitting is reasonable (Figure 2b). However, no electron density was visible for the benzoic group in the mid-region of the fluorophore molecule, indicating that this region is more disordered as compared to other parts of the fluorophore molecule.

In our PenP-E166Cf structure the fluorescein molecule partially occupies the outer edge of the antibiotic binding region and is in close contact with several residues at the catalytic site. The maleimide moiety near the thiol linkage site is inserted into the catalytic core, located within 2.5 Å from the side chain of Ser70 on one side and 3.5 Å away from Ω-loop on the other side. The xanthene group near the other end of the fluorescein molecule extends toward the solvent (Figure 2b), loosely packed against β-strand B3 that forms part of the extended substrate binding area involved in coordinating antibiotics as shown in the extended-spectrum class A *β*-lactamase, Toho-1, in complex with cefotaxime, cephalothin, and benzylpenicillin [19]. No specific interactions were observed between fluorescein and the protein. Total solvent accessible area is 188 Å^2^, 33% of the total surface area, indicating that fluorescein is partially packed against the PenP molecule and not fully solvent exposed. The fluorescein molecule is highly dynamic, as reflected by the poor electron density map as well as high average temperature factor (~72.3). In contrast, the rest of the structure shows excellent electron density and low average temperature factor (~23.5) that is typical of the 2.2 Å data set. The Ω-loop, on which the fluorescein molecule is conjugated, was little affected by the dynamic fluorophore and adopts the same conformation as that of the unlabelled PenP (Figure 2b).

### The structure of PenP-E166Cf in complex with cefotaxime

We chose to determine the PenP-E166Cf-cefotaxime structure, using cefotaxime as a representative of the many β-lactam antibiotics because of its positive fluorescence response induced in PenP-E166Cf as well as its chemical structure that contains functional groups typical of both second- and third-generation antibiotics. Cefotaxime was soaked into the PenP-E166Cf crystals by incubating the crystals in the reservoir solution with 0.01 M cefotaxime added for 20 minutes. The PenP-E166C structure, without the conjugated fluorescein molecule, was used as the starting model for structure determination. After initial rounds of refinement both the *fo-fc *and 2*fo-fc *electron density maps were carefully inspected for evidence of cefotaxime and fluorescein, as well as for any structural changes on PenP.

**Figure 3:**
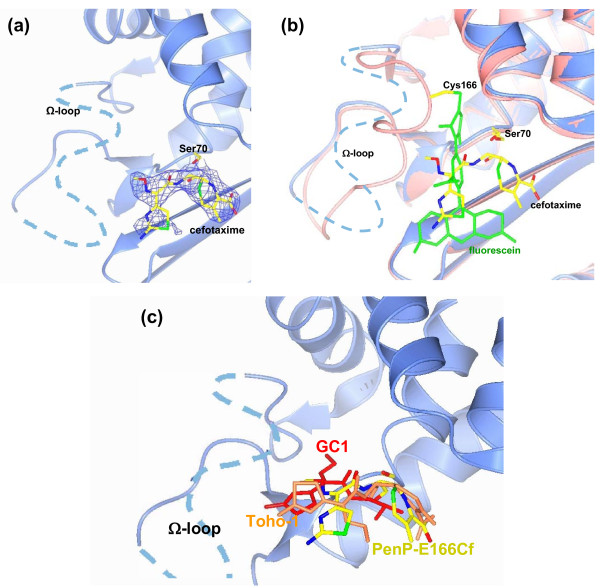
**Crystal structure of PenP-E166Cf-cefotaxime**. (a) The *fo-fc *map of cefotaxime in PenP-E166Cf-cefotaxime complex contoured at 2.0 σ. The light blue dash line represents the disordered Arg164 to Pro174 due to the poor electron density. (b) Comparison of PenP-E166Cf-cefotaxime complex with *apo *PenP-E166Cf structure. The two structures are superimposed by main chain atoms. Key residues including Cys166, Ser70 and cefotaxime are also shown in cpk cylinder model. (c) Comparison of binding mode of cefotaxime in PenP-E166Cf with that of Toho-1 and GC-1. PenP-E166Cf, Toho-1 and GC1 are superimposed by aligning on overall main chain atoms. Cefotaxime is in cylinder model colored in cpk (PenP-E166Cf-cefotaxime), golden (Toho-1) and red (GC1) respectively.

The cefotaxime was clearly visible in *fo-fc *electron density map as covalently bonded through its carbonyl carbon atom C7 to the O^γ ^atom of Ser70, which represents the acylated ES* adduct (Figure 3a). But we could not identify any electron density in either *fo-fc *or 2*fo-fc *map that would be accountable for fluorescein molecule around the location seen in PenP-E166Cf or anywhere nearby. Furthermore, the *fo-fc *map showed strong negative signal for a large segment of Ω-loop (residues 164 to 174) and the 2*fo-fc *map showed no electron density for this region at all, indicating this region became highly disordered upon acylation of cefotaxime (Figure 3b). Based on these observations we did not include fluorescein molecule or the disordered region of Ω-loop in our final refined structure of PenP-E166Cf-cefotaxime.

The overall structure folding of fluorescein-labeled and cefotaxime-bound PenP is nearly identical to that of the wild-type unlabeled PenP and the fluorescein-labeled PenP-E166Cf. From the calculation result by the CCP4 program, it was found that the B factor of Glu163, Gly175, Glu176 on Ω-loop, which are next to the disordered region, is significantly higher (~65 Å^2^) than other parts of the protein (~20 Å^2^). The refinement statistic for this set of crystal structure has different values from that of the apo PenP-E166Cf structure due to the cefotaxime and the difference in Ω-loop.

To investigate why the binding and acylation of cefotaxime causes the Ω-loop and the conjugated fluorescein molecule to become highly flexible and structural disordered, we superposed the PenP-E166Cf structure onto the PenP-E166Cf-cefotaxime complex structure. Fluorescein is seen as occupying a site that partially overlaps with the acylated cefotaxime; particularly the benzoic group of fluorescein molecule is in direct steric clash with the 7-amino substituent of cefotaxime (Figure 3b). Thus the binding and acylation of cefotaxime to PenP would displace fluorescein from its original position to avoid steric clash. It is likely that the Ω-loop, in order to accommodate such displacement, loses its well-folded structure and becomes highly flexible. As a consequence the fluorescein molecule conjugated to the flexible Ω-loop becomes fully exposed to the polar aqueous environment, leading to enhanced fluorescence. Thus our structural findings confirmed our initial proposal of a biosensing mechanism based on displacement of fluorescein [15,16].

To understand the impact of conjugated fluorescein molecule on the substrate binding kinetics of PenP we compared the PenP-E166Cf-cefotaxime structure to two other *β*-lactamase structures in complex with cefotaxime, including the narrow-spectrum Toho-1 and the extended-spectrum GC1 [19,20]. In Toho-1 structure the methoxyimino side chain points away from the active site and is solvent-exposed (Figure 3c). Such an orientation packs the methoxyimino side chain tightly against the thiozolyl ring, leading to a distorted configuration of the cephem nucleus that is catalytically incompetent for deacylation [19]. In GC1 structure the transition analog of cefotaxime binds to GC1 in a fully extended conformation, with oxyimino group inserted to active site and extended away from the thiozolyl ring (Figure 3c). This conformation is regarded as catalytically competent to facilitate deacylation because the distortion on the cephem nucleus is released [20]. Importantly, the binding mode of cefotaxime in our PenP-E166Cf-cefotaxime structure closely resembles that of GC1 (Figure 3c), suggesting that with its Ω-loop fully flexible the naturally narrow-spectrum PenP can accommodate cefotaxime in a manner that resembles the extended-spectrum GC1.

## Conclusions

Our structural studies indicate the molecular mechanism how fluorescein-labeled *β*-lactamase detects *β*-lactam antibiotics. The conjugated fluorescein molecule is located near the catalytic site and partially occupies the antibiotic binding region. The binding and acylation of *β*-lactam antibiotics such as cefotaxime would expel the fluorescein molecule from its original position and leads to increased flexibility of the Ω-loop, to which the fluorophore is linked. As a result, the fluorophore is relocated from its original position with partial solvent exposure to become fully solvent exposed, leading to enhanced fluorescence emission. These findings confirm our previous proposal based on structural modeling.

Furthermore the Ω-loop demonstrates the propensity of becoming highly flexible and unstructured if its tight packing against the catalytic site is disturbed. Such increased flexibility enables PenP to bind and acylate cefotaxime, a naturally poor substrate, in a manner that resembles the extended-spectrum cefotaxime-resistant *β*-lactamases. This finding could be valuable in the future design of novel antibiotics that resist the binding or hydrolysis by β-lactamases.

## Methods

### Protein expression and purification

Two constructs of PenP protein were used for our experiments, the maltose binding protein (MBP)-fusion construct for time-dependent fluorescence measurements and the His_6_-tagged construct for crystallization and structural studies, as well as scanning fluorescence spectra. The MBP fusion has been shown not to interfere with fluorescence measurements in our previous studies (data not shown). The MBP-fusion construct was cloned into pMAL-c2X vector (NEB). The His_6_-tagged PenP enzyme was cloned into a modified pRset-A vector (Invitrogen) with a *TEV *protease cleavage site upstream of the PenP gene. The E166C mutation was constructed using QuikChange Site-Directed Mutagenesis Kits (Strategene).

The MBP-fusion construct was expressed in *E. coli *strain BL21 (DE3) at 37°C for overnight after induction by 300 μM IPTG when A_600 _reached 0.5-0.7. The harvested cells were centrifuged and lysed by sonication. The supernatant after sonication was passed through amylose affinity chromatography. The eluted fractions were pooled and buffer exchanged to 20 mM ammonium bicarbonate. The protein was freeze-dried for storage afterwards.

The His_6_-tagged PenP protein was expressed in *E. coli *strain BL21 (DE3) at 37°C for overnight after induction by 200 μM IPTG when A_600 _reached 0.8-1.2. The harvested cells were centrifuged and the supernatant was passed through Nickel affinity chromatography, followed by DEAE anion exchange chromatography. The fractions containing the target protein were pooled and concentrated by Amicon^® ^Ultra-15 Centrifugal Filter Devices (Millipore NMWL = 10,000). The His_6_-tag was cleaved by adding the *TEV *protease in 1:20 molar ratio to the concentrated PenP-E166C protein (2 mg/ml). The mixture was incubated at 30°C for 6 hours and was further purified by Nickel affinity chromatography to remove uncleaved protein.

### Fluorescein labeling of PenP-E166C to form PenP-E166Cf

A ten-fold molar excess of fluorescein, with concentration of 20 mM, was dissolved in DMF (Dimethyl formamide) and added to the concentrated PenP-E166C protein solution drop by drop. The labelling reaction was allowed to proceed in darkness with stirring for 1 hour, and then dialysed against 50 mM potassium phosphate buffer (pH 7.0) at 4°C for several times in order to remove excess fluorescein. The labelled PenP-E166Cf protein was concentrated to less than 1 ml and further purified by Superdex™ 75 gel filtration column (GE Healthcare). The running buffer contains 20 mM Tris-HCl, 50 mM NaCl, pH 7.5. The target fractions were pooled and concentrated by Amicon Ultra to 25 mg/ml. The labelling efficiency was confirmed by ESI-MS.

### Fluorescence spectra of PenP-E166Cf for antibiotic detection

Fluorescence profile of PenP-E166Cf alone, as well as in presence of various *β*-lactams were measured using Perkin-Elmer LS50B spectrofluorimeter. Both scanning spectra and time-dependent spectra were measured. Different *β*-lactam antibiotics, including cefotaxime, ceftriaxone, ceftazidime, cephaloridine, cephalothin, cefoxitin, cefuroxime, penicillin G, and ampicillin, were incubated with PenP-E166Cf for 100 s at 1 μM to allow sufficient acylation of the antibiotic to form ES* adduct. The product after acylation was subjected to fluorescence measurement as previously described [15].

### Crystallization, structure determination and refinement

Crystals of PenP-E166Cf were grown by hanging-drop vapour diffusion method after mixing 1 μl of protein and 1 μl of reservoir solution containing 25% (w/v) PEG 4000, 0.1 M Hepes pH 7.2, 0.4 M NH_4_Acetate and 0.2 M K_2_HPO_4_. Small crystals in the form of clustered needles appeared readily. For data collection, single crystals were obtained after separating them from the clustered needles. Crystals were harvested and cryoprotected in its reservoir solution supplemented with 20% ethylene glycol for one minute prior to flash freeze and data collection on the Rigaku MicroMax™-007HF x-ray machine. For PenP-E166Cf-cefotaxime data set, crystals were soaked in its growth solution added with 0.01 M of cefotaxime for 15 minutes and then mounted to the x-ray machine. Data were integrated and scaled by CrystalClear™1.3.5 SP2 (Rigaku Inc.).

The crystals belong to the monoclinic group P2_1 _with cell parameter: a = 43.43 Å, b = 92.3 Å, c = 66.43 Å and β = 104°. The PenP-E166Cf crystals diffracted to 2.15 Å resolution, while the PenP-E166Cf-cefotaxime crystal diffracted to 2.8 Å. Both structures were determined by molecular replacement using PenP structure as the search model (PDB ID 4BLM) [21]. The program COOT was used for inspection of electron density maps and model building [22]. There are two molecules per asymmetric unit. The fluorescein and cefotaxime molecules were built by PRODRG [23] and appended to the PenP structure for refinement. Structure determination and refinement of PenP-E166Cf and PenP-E166Cf-cefotaxime were done using the CCP4 program suite [18]. A summary of the crystallographic data and refinement statistics are given in Table 1. The coordinates and structure factors from this study have been deposited into Protein Data Bank (PDB) under accession codes 3M2J (PenP-E166Cf apo structure) and 3M2K (PenP-E166Cf-cefotaxime).

**Table 1:** X-ray data-collection and structure refinement statistics.

	E166Cf	E166Cf+cefotaxime
*PDB code*	3M2J	3M2K

		

*Data collection*		

Space group	*P*2_1_	*P*2_1_

Unit cell parameters (Å)		

*a*	43.3	43.5

*b*	92.3	91.4

*c*	66.3	66.1

*β*	104.82	104.52

Resolution range (Å)	52-2.15 (2.24-2.15)	45-2.80 (2.95-2.80)

No. of total reflections	79750	40611

No. of unique reflections	29537	12412

*I/σ*	7.1 (2.7)	6.3 (2.4)

Completeness (%)	97.0 (99.5)	99.8 (99.9)

*R*_merge _(%)	9.7 (27.1)	11.8 (32.0)

		

*Structure refinement*		

Resolution (Å)	50.0-2.20	45.0-2.80

*R*_cryst_/*R*_free _(%)	20.0/23.2	21.2/27.7

r.m.s.d. bonds (Å)/angles (°)	0.018/1.784	0.010/1.672

No. of reflections		

Working set	24217	11749

Test set	1291	647

No. of atoms		

Protein atoms	4011	3706

Water molecules	254	29

Average *B*-factor (Å^2^)		

Main chain	24.7	16.96

Ligand molecules	48.4	42.46

Water	32.7	10.7

## Authors' contributions

WTW performed experiments, analyzed data and drafted manuscript. HWA and HKY assisted in experiments. YXZ, KYW and YCL designed project, analyzed data and drafted manuscript. All authors read and approved the final manuscript.
